# Cellulose filtration of blood from malaria patients for improving ex vivo growth of *Plasmodium falciparum* parasites

**DOI:** 10.1186/s12936-017-1714-2

**Published:** 2017-02-10

**Authors:** Sixbert I. Mkumbaye, Daniel T. R. Minja, Jakob S. Jespersen, Michael Alifrangis, Reginald A. Kavishe, Steven B. Mwakalinga, John P. Lusingu, Thor G. Theander, Thomas Lavstsen, Christian W. Wang

**Affiliations:** 1 Kilimanjaro Clinical Research Institute, Kilimanjaro Christian Medical University College, Moshi, Tanzania; 2 Korogwe Research Station, Tanga Centre, National Institute for Medical Research, Tanga, Tanzania; 3 Department of Immunology and Microbiology, Faculty of Health and Medical Sciences, Centre for Medical Parasitology, University of Copenhagen, CSS Building 22-23, Øster Farimagsgade 5, PO Box 2099, 1014 Copenhagen K, Denmark; 4 Department of Infectious Diseases, Rigshospitalet, Copenhagen, Denmark

**Keywords:** *Plasmodium falciparum*, Cellulose column filtration, Ex vivo growth

## Abstract

**Background:**

Establishing in vitro *Plasmodium falciparum* culture lines from patient parasite isolates can offer deeper understanding of geographic variations of drug sensitivity and mechanisms of malaria pathogenesis and immunity. Cellulose column filtration of blood is an inexpensive, rapid and effective method for the removal of host factors, such as leucocytes and platelets, significantly improving the purification of parasite DNA in a blood sample.

**Methods:**

In this study, the effect of cellulose column filtration of venous blood on the initial in vitro growth of *P. falciparum* parasite isolates from Tanzanian children admitted to hospital was tested. The parasites were allowed to expand in culture without subcultivation until 5 days after admission or the appearance of dead parasites and parasitaemia was determined daily. To investigate whether the filtration had an effect on clonality, *P. falciparum* merozoite surface protein 2 genotyping was performed using nested PCR on extracted genomic DNA, and the *var* gene transcript levels were investigated, using quantitative PCR on extracted RNA, at admission and 4 days of culture.

**Results:**

The cellulose-filtered parasites grew to higher parasitaemia faster than non-filtered parasites seemingly due to a higher development ratio of ring stage parasites progressing into the late stages. Cellulose filtration had no apparent effect on clonality or *var* gene expression; however, evident differences were observed after only 4 days of culture in both the number of clones and transcript levels of *var* genes compared to the time of admission.

**Conclusions:**

Cellulose column filtration of parasitized blood is a cheap, applicable method for improving cultivation of *P. falciparum* field isolates for ex vivo based assays; however, when assessing phenotype and genotype of cultured parasites, in general, assumed to represent the in vivo infection, caution is advised.

**Electronic supplementary material:**

The online version of this article (doi:10.1186/s12936-017-1714-2) contains supplementary material, which is available to authorized users.

## Background

Malaria continues to be a major cause of human suffering and death [1]. Children under the age of five and pregnant women infected with the parasite *Plasmodium falciparum* are the worst affected. The current first-line drug of choice, artemisinin and its derivatives, has been shown to be a powerful tool in combatting malaria; however, the spread of resistant *P. falciparum* parasites in Southeast Asia poses a serious threat to communities in malaria-endemic areas [2, 3].

Individuals in endemic areas develop immunity to severe malaria by acquiring antibodies that inhibit the parasite’s ability to invade erythrocytes and adherence of infected erythrocytes to endothelial receptors of the microvasculature [4]. Malaria pathogenesis is tied to parasite sequestration in host organs and therefore, the parasite adhesion proteins, *P. falciparum* erythrocyte membrane protein 1 (PfEMP1)-mediating sequestration, are major targets for acquired immunity [5].

For studies of geographic variations of anti-malarial drug sensitivity and mechanisms of pathogenesis and immunity, establishment of phenotypes of the parasites causing infection in individual patients is important. This is best done by establishing in vitro *P. falciparum* culture lines from parasites isolated from patients. However, in vitro culturing of parasites collected by venepuncture from patient blood is not straightforward. Leucocytes and platelets can adversely affect parasite development by phagocytosis and degranulation [6, 7]. Cellulose column filtration of blood is an inexpensive, rapid and effective method for the removal of these host factors and has improved cultivation of *Plasmodium vivax* [8, 9]. Furthermore, filtration reduces the amount of human DNA and makes full genome sequencing of parasites an affordable task [10].

In this study, the effect of cellulose column filtration of venous blood on the initial in vitro *P. falciparum* growth, clonality and *var* gene expression of *P. falciparum* parasites from Tanzanian children admitted to hospital with malaria was tested.

## Methods

### Sample collection

Children, under the age of 5 years, admitted at the paediatric ward of Korogwe District Hospital, in northeastern Tanzania, were enrolled as part of an ongoing monitoring of malaria at the hospital. Children were immediately subjected to a clinical investigation, and a blood sample was collected for diagnostic and research purposes. The children were first tested by malaria rapid diagnostic test (RDT) followed by examination of thick and thin blood smears. During a 4-week period in May–June 2016, venous blood samples from 13 children, blood smear-positive for *P. falciparum* were collected (Additional file 1). All samples with a parasitaemia above 0.4% (15,000 parasites/µL, n = 10), were further processed for in vitro parasite culturing. In the course of parasite culture three samples were discarded due to bacterial contamination in both cellulose-filtered and non-filtered cultures, leaving seven parasite cultures for analysis.

### Parasite filtration and culturing conditions

Venous blood samples were collected in 6-mL citrate phosphate dextrose adenine (CPDA) tubes (Greiner Bio-One, Austria) and processed immediately. Blood was centrifuged for 5 min at 800×*g*, plasma removed and an equal volume of RPMI-1640 (Sigma Life Sciences) was added to the erythrocytes and buffy coat and gently mixed. The mixture was gently layered on top of 4 mL Lymphoprep™ (StemCell Technologies Inc, Axis-Shield PoC AS, Oslo, Norway), centrifuged at room temperature for 20 min at 800×*g* with the brake off. Supernatant was discarded and the peripheral blood mononuclear cells (PBMC) were removed and stored. Two-hundred µL of packed erythrocytes, solely infected with ring stage parasites, were added to 2 mL Trizol (Invitrogen) to preserve RNA, and 200 µL was added to an equal volume of freezing medium (sterile filtered 3% d-sorbitol, 28% glycerol and 0.65% NaCl) and snap frozen in liquid nitrogen to preserve ring stage parasites. A portion was put into a total of 100 µL type O^+^ erythrocyte culture (T25 culture flask, SPL Life Sciences) with approximately 0.2% parasitaemia and RPMI-1640 supplemented with Albumax II, as previously described [11], at a 1% haematocrit. Culture flasks were gassed for 30 s (2.4% O_2_, 5.4% CO_2_, 92.2% N_2_) and incubated at 37 °C. Initial parasitaemia was determined by assessment of 2000 erythrocytes on thin smears stained by Giemsa.

The remainder of the erythrocytes, 0.5–1.5 mL, was filtered through a cellulose-packed column to remove leucocytes and platelets as previously described [8, 9], with the following modifications: the column, a 10-mL syringe (BD-Luer-Lock™ Tip) was packed with 3 mL cellulose fibre (Cat No. C6288, Sigma-Aldrich), fitted with two layers of Whatmann™ lens paper (GE Healthcare Life Sciences) covering the outlet. The column was then autoclaved and stored clean and sterile prior to use. Upon use, the cellulose was wetted in pre-heated 37 °C sterile phosphate buffered saline (PBS) allowing it to run through the syringe, and infected erythrocytes (IEs), diluted in equal volume of PBS, were added and gently pressed to run through in droplets. The sample was washed with PBS and the filtrate was centrifuged for 8 min at 800×*g*; supernatant was discarded and parasites were cultured as described for the unfiltered parasites, aiming at approximately 0.2% parasitaemia determined by assessment of 500 erythrocytes on thin smears stained by Giemsa. The developmental stage (ring and/or late stage) and parasitaemia were determined for the non-filtered and filtered cultures, respectively, every 18–24 h by assessment of minimum 500 erythrocytes, until the appearance of dead parasite in the culture or 5 days after initial blood sampling at admission without subcultivation. On the fourth day, 50 µL IEs were pelleted and 1 mL Trizol was added to preserve RNA.

### RNA and quantitative PCR

RNA was extracted using Trizol (Invitrogen) according to the manufacturer’s instructions. Samples were treated with DNase I (Sigma) to digest any genomic DNA and tested in quantitative PCR (qPCR) for contamination, using a primer set for the *seryl*-*tRNA synthetase* gene [12]. RNA was reverse transcribed from random hexamers, using Superscript II (Invitrogen), according to the manufacturer’s instructions (Invitrogen). Transcript levels of *var* gene subtypes were generated using a set of 12 qPCR primers targeting loci specific for different encoded PfEMP1 domains as previously described [13, 14]. qPCR was performed in 20-μL reactions using QuantiTect SYBR Green PCR master mix (Qiagen) with the Rotorgene thermal cycler system (Corbett Research), and the transcript level was determined relative to the averaged transcript level of *seryl*-*tRNA synthetase* and *fructose*-*bisphosphate aldolase*, as previously described [13].

### DNA extraction and quantification of human versus parasite DNA

Cellulose filtration was done for all collected samples regardless of parasitaemia and approximately 100 µL of filtered IEs were saved at −20 °C for the extraction of gDNA. DNA was extracted using the DNeasy Kit (Qiagen) according to the manufacturer’s instructions and was tested in qPCR using a primer set for the *P. falciparum seryl*-*tRNA synthetase* gene and a primer set for the human *beta*-*2*-*microglobulin* housekeeping gene B2M-F1 TTGACAGGATTATTGGAAATTTGTT B2M-R1 TAACCACAACCATGCCTTACTTTA as above. The difference in copy numbers was calculated as the ΔCt method, and normalized using the size of the human haploid genome (~3.2 billion bp) and *P. falciparum* genome (~23 million bp).

### Genotyping

*Plasmodium falciparum* merozoite surface protein 2 (*Pfmsp2*) genotyping was performed on extracted gDNA from parasites at day 0 and at day 4, as previously described [15], with the following modifications: the conditions of the outer PCR were as follows: 94 °C for 15 min, 30 cycles of 94 °C for 1 min, 58 °C for 2 min and 72 °C for 2 min, followed by 58 °C for 2 min and 72 °C for 5 min; for the nested PCR reactions: 94 °C for 15 min, 30 cycles of 94 °C for 30 s, 61 °C for 1 min and 72 °C for 1 min, followed by 58 °C for 2 min and 72 °C for 5 min. The PCR reactions contained 0.00625 µM of the outer primer pairs included in the outer PCR and 0.125 μM of the primer pairs for the nested PCRs, 1:1 of TEMPase Hot Start DNA Polymerase (Ampliqon, VWR) and 1 μL of purified DNA or PCR product. A set of the most common, in-house, used laboratory isolates (3D7 and HB3) as well as *P. falciparum* negative controls were included in the set-up and the amplified PCR products were analysed by agarose gel electrophoresis.

### Statistical analysis

To compare the total parasitaemia between the cellulose-filtered parasites and non-filtered parasites through the 5 days of culturing, generalized least squares (GLS) random-effects regression model was used to calculate statistical significance. To compare the parasite development through the different life-cycle stages between the cellulose-filtered parasites and non-filtered parasites, Wilcoxon’s paired signed rank test was used to calculate statistical significance. Statistical analysis was performed using Stata Statistical Software 14.2. A *P* value below 0.05 was considered statistically significant.

## Results

### In vitro growth of cellulose-filtered versus non-filtered *Plasmodium falciparum* isolates

**Fig. 1:**
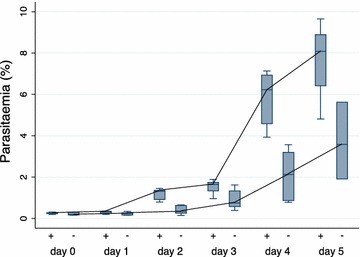
Parasitaemia of *Plasmodium falciparum* parasites in culture. *Box plots* showing the median parasitaemia (%) recorded for every 24 h from day 0 to day 5 of in vitro culture of seven patient parasite isolates either with prior cellulose filtration (+) or no filtration (−). The *box plots* display median, 1st quartile (Q1), and 3rd quartile (Q3), and the *lower whisker* indicates the lowest data point above Q1 minus 1.5 times IQR; the* upper whisker* indicates the highest data point below 1.5 IQR plus Q3. Cellulose filtration was a positive predictor for parasitaemia calculated using generalized least squares (GLS) random-effects regression model (P < 0.001). The growth profile is highlighted with a line through the medians

**Fig. 2:**
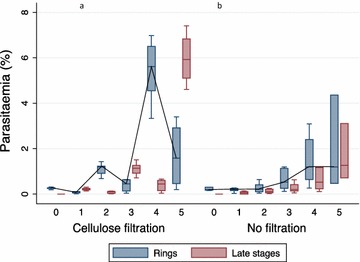
Parasitaemia of ring- and late-stage parasites in **a** cellulose-filtered and **b** non-cellulose-filtered parasites. *Box plots* showing the median parasitaemia (%) of ring- and late-stage parasites recorded for every 24 h from day 0 to day 5 of in vitro culture of seven patient parasite isolates either with prior cellulose filtration (+) or no filtration (−). The *box plots* display median, 1st quartile (Q1), and 3rd quartile (Q3) and the *lower whisker* indicates the lowest data point above Q1 minus 1.5 times IQR, the *upper whisker* indicates the highest data point below 1.5 IQR plus Q3. The growth profile is highlighted with a line only for the ring-stage parasites through the medians

The effect of cellulose filtration on the initial in vitro growth of *P. falciparum* was tested. Following Lymphoprep™ (StemCell Technologies Inc, Axis-Shield PoC AS, Oslo, Norway) treatment, IEs from seven patients were adjusted to approximately 0.2% parasitaemia and cultured with and without prior cellulose filtration. Parasitaemia and developmental stage (ring and/or late stage) was registered every day for each culture through a minimum of two intra-erythrocytic life cycles (Additional file 2). On day of admission, as expected, only ring-stage parasites were observed. It was evident that the cellulose-filtered parasites grew to higher parasitaemia faster than the non-filtered parasites (P < 0.001) (Fig. 1). The non-filtered parasites showed a more mixed composition of ring- and late-stage parasites throughout the experiment (Fig. 2b), whereas the cellulose-filtered were more synchronous (Fig. 2a).

**Fig. 3:**
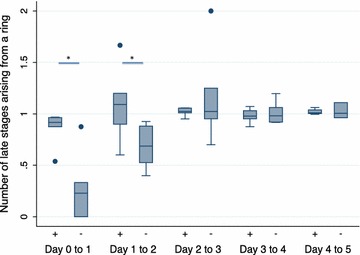
Number of ring-stage parasites that develop into late stages. *Box plots* showing the number of parasites developing from ring- to late-stage parasites, either cellulose-filtered (+) or non-filtered parasites (−) from day 0 to day 5. The *lower whisker* indicates the lowest data point above Q1 minus 1.5 times IQR, the* upper whisker* indicates the highest data point below 1.5 IQR plus Q3. Values below Q1 minus 1.5 IQR and above Q3 plus 1.5 IQR are indicated as points. Statistical significance was calculated using Wilcoxon’s signed rank test. *P = 0.02

During the first 24 h of culture, the proportion of parasites developing from ring to late stage was significantly higher among cellulose-filtered parasites than among the non-filtered parasites (P = 0.02) with an average of 87% of the filtered ring stages developing into late stages compared to only 28% of the non-filtered parasites (Fig. 3). After 48 h, an average of 71% of the non-filtered ring stages observed at 24 h had progressed into late-stage parasites (P = 0.02). Thereafter, no difference was found between filtered and non-filtered parasites, with an average transformation rate around 100%.

The in vitro parasite multiplication rate, calculated as the number of ring stages observed compared to the number of late stages observed the previous day, was around 4.8 for all cultures with no significant difference between filtered or non-filtered parasites or days post admission (Additional file 3).

### Removal of human DNA

In a *P. falciparum*-infected whole blood sample, human leucocyte DNA typically represents more than 99% of the total DNA. To test the amount of human and *P. falciparum* DNA in the blood after cellulose filtration, gDNA was extracted and tested by qPCR, using human and *P. falciparum*-specific primer set targeting *beta*-*2*-*microglobulin* and *seryl*-*tRNA synthetase*, respectively, on all 13 collected samples (Table 1). In all samples the parasite gene was found in higher copy numbers than the human gene (Table 1). In three samples (4636, 4659, 4661), human DNA seemed to make up more than 50% of the total human and parasite DNA, when accounting for the difference in size of the genomes. gDNA from erythrocytes subjected to Lymphoprep™-treatment only was also extracted (n = 7). When comparing human and parasite DNA in the Lymphoprep™-treated portion of the sample, with the portion that was both Lymphoprep™-treated and cellulose-filtered, there was a clear increase in the parasite versus human DNA ratio in five of the seven samples when cellulose filtration was applied, irrespective of initial parasitaemia (Table 1; Additional file 1).

**Table 1:** Parasite versus human DNA in Lymphoprep™ and cellulose-filtered, and Lymphoprep™-treated only samples

ID	Lymphoprep™ and cellulose-filtered			Lymphoprep™-treated only		
	Ct *P*. *f*.	Ct Human	% *P*. *f*. DNA	Ct *P*. *f*.	Ct Human	% *P*. *f*. DNA
4636	15.8	18.9	6	11.6	24.1	98
4638	18.7	ND	100	15.3	29.9	99
4647	14.5	33.7	100			
4648	15.8	33.8	100			
4649	17.1	32.8	100			
4651	13.6	33.4	100			
4656	15.4	ND	100			
4658	12.1	21.0	77	15.8	20.6	16
4659	17.4	18.2	1	19.4	21.4	3
4660	12.4	21.5	80	15.8	22.3	39
4661	26.5	32.9	38			
4662	18.5	31.0	98	21.0	25.0	10
4663	18.7	ND	100	16.2	24.5	69

Ct-value for *seryl*-*tRNA*-*synthetase* and *beta*-*2*-*microglobulin* (*b2m*) specific primer pairs, fold change calculated as dCt between *seryl*-*tRNA*-*synthetase* and *b2m*, then normalized by the difference in genome size

% *P. falciparum* DNA in a sample compared to human DNA

*ND* none detected

### *Plasmodium falciparum msp2* genotyping

Results from PCR *msp2* genotyping showed that the children were infected with a mixture of several *P. falciparum* clones at admission (mean number of clones: 3.7, range 2–5 *P. falciparum* clones/infection) (Table 2). There was no considerable difference in size and number of amplified *msp2* IC1 and FC27 DNA fragments when samples were extracted from filtered and non-filtered parasites either at admission or after 4 days of culture (Table 2). However, when comparing allele profiles at admission with the profile after 4 days of culture, some dominant alleles were absent after only two intra-erythrocytic developmental cycles, while other alleles, not initially detected, dominated the parasite culture.

**Table 2:** *Msp2* genotyping for the seven parasite isolates

ID	Sampling	IC1	FC27	Clones
4636	Admission	AB	AB	4
	Cellulose filtration	AB	AB	4
	4 days culture	C	CD	3
	4 days culture CF	AD	CDEF	6
4638	Admission	AB	AB	4
	Cellulose filtration	A(B)	A	3
	4 days culture	0	C	1
	4 days culture CF	0	CDE	3
4658	Admission	AB	A	3
	Cellulose filtration	AB	0	2
	4 days culture	(B)CD	BC(DEF)	8
	4 days culture CF	(B)C	BCD(EF)	7
4659	Admission	A(B)	A(BC)	5
	Cellulose filtration	AB	(A)	3
	4 days culture	(B)CD	CDE	6
	4 days culture CF	(B)CD(E)	CDE	7
4660	Admission	AB	0	2
	Cellulose filtration	AB	A	3
	4 days culture	A	ABC	4
	4 days culture CF	(C)	ABC	4
4662	Admission	0	A(BC)	3
	Cellulose filtration	0	A(BC)	3
	4 days culture	A(B)	A(BC)	5
	4 days culture CF	A	AB(CD)	5
4663	Admission	AB	ABC	5
	Cellulose filtration	0	A	1
	4 days culture	A(CD)	CDE(F)	7
	4 days culture CF	AC	CDE(F)G	7

*Msp2* genotyping for the seven parasite isolates, with primers for the two IC1 and FC27 allele families at admission, after filtration, 4 days of culture with no cellulose filtration, and at 4 days of culture with prior cellulose filtration (CF). A, B, C, D, E, F, G designate different observed allele sizes within each allele family and in brackets () if an uncertainty of the band exists. 0 = no alleles observed

### *Var* transcript analysis

**Fig. 4:**
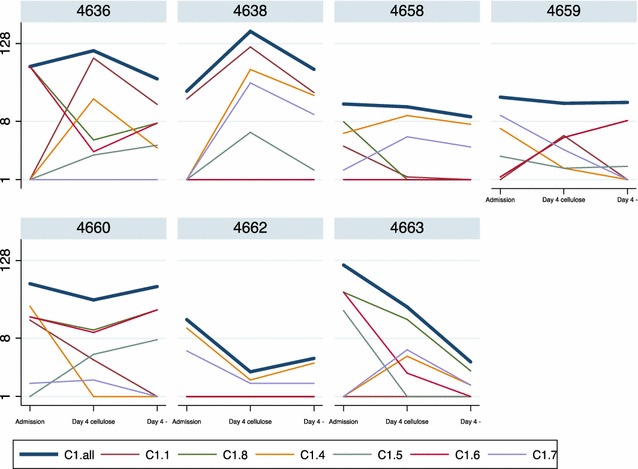
*Var* transcript levels at admission and after 4 days of culture. Transcript levels (shown as transcript units) of six CIDRα1 *var* subtypes in *P. falciparum* parasites at admission, after 4 days in culture of the cellulose-filtered parasites, and after 4 days in culture of the non-filtered parasites. CIDRα1.all is a summated transcript level for all the CIDRα1-domain primers, as described in [14]. C1.all = CIDRα1.all; C1.1 = CIDRα1.1; C1.8 = CIDRα1.8; C1.4 = CIDRα1.4; C1.5 = CIDRα1.5; C1.6 = CIDRα1.6; C1.7 = CIDRα1.7

Transcript levels of *var* genes were quantified. *Var* genes encode PfEMP1 proteins that are expressed on the surface of IEs and mediate sequestration in the vascular tissue. A panel of quantitative PCR primers targeting different cysteine-rich interdomain region (CIDR) *var* gene loci was used. The primer sensitivity and specificity is described in [14] and in general the primers had good coverage of sequence variants of CIDRα1-domains predicted to bind EPCR [16] and the conserved VAR2CSA and VAR3 (Additional file 4). The median transcript levels did not change noticeably in parasites from admission and 4 days of culture, regardless whether parasites had been filtered or not (Additional file 4). However, the transcript levels of specific CIDRα1-subtypes within patient parasite isolates changed substantially between admission and 4 days of culture, for both filtered and non-filtered parasites, but in no predictable direction, yet the overall CIDRα1-level (CIDRα1.all) did not change significantly (Fig. 4). There was no association between the change in *msp2* genotype and change in *var* type transcript levels.

## Discussion

Ensuring the growth of ex vivo parasite isolates is a fundamental requirement for investigating phenotypes of the parasites causing an infection. Here, it was shown that ex vivo parasites that are filtered through a cellulose-column before cultivation have higher growth rates and show a more synchronous profile in culture than without the filtration during the initial stages of cultivation. This was found to be mainly due to a higher rate of ring stages progressing into late stages in the first 48 h of culture. The reason for the halting development of those parasites not cellulose-filtered is best explained by the removal of human components from the culture. It was expected that removal of leucocytes and platelets optimize the survival of cultivated parasites [6, 7]. As cellulose-filtration increases the removal of human DNA compared to Lymphoprep™-treatment alone, this could explain the higher growth rates. However, the increase in parasite DNA versus human DNA was found in only five out of the seven patient parasite isolates, and may not represent the only reason for higher growth rates. It could be speculated that other unknown growth-inhibiting components also are removed.

Despite being based on only seven patient parasite isolates, the current study suggested an increased success in establishing in vitro growth of parasite isolates when using cellulose-filtered blood samples. Substituting Albumax II with heat-inactivated human serum to the culture may add further advantage for establishing long-term cultures. Furthermore, cellulose filtration prior to cryopreservation of field isolates could potentially increase growth rates of the later thawed parasites but this was not investigated.

 The *msp2* profile of the two families, IC1 and FC27, was examined and showed that cellulose filtration had no significant effect on the amplified genotypes. However, after only two intra-erythrocytic developmental cycles in vitro, the profiles differed substantially from initial cultivation. This could to be due to some parasites adapting more easily to culture than others, and therefore outgrowing the initial dominant genotypes. Others have shown that short- and long-term cultures of field isolates drastically fluctuate in genetic profile, displaying a constant appearance, disappearance and re-appearance of parasite clones, and speculate that cultured parasites are not an accurate representation of the natural parasite genetic in circulation [17]. Furthermore, an earlier study in Tanzania showed considerable in vivo day-to-day variation in *msp1* and *msp2* allele profiles between samples collected from the same child on successive days, followed for 31 days [18]. Färnert et al. [19] also found appearance, disappearance and re-appearance of strains and individual clones in natural infection over time, which was shown to occur in as few as 6-h intervals. Thus, the in vitro results may reflect natural in vivo fluctuations in genotype prevalence. Such fluctuation could drastically affect the phenotype of the cultured parasites, although Yeda et al. [17] did not find a correlation between the phenotypic and genotypic changes when looking at drug resistance markers. Here, the *var*-transcript profiles of the parasites at admission and after 4 days of culture were investigated. Extensive changes in *var* transcript levels reported by single primer sets specific for different CIDRa1-domain types were observed. However, these changes could not be explained by the changes in prevalent genotype. Differences in *var* transcript-levels may also be affected by the differences in the time-point of sampling and the ratio of ring- to late-stage parasites, as *var* transcript levels change during the erythrocytic development with transcript-levels peaking in ring-stage parasites [12, 20]. This could not be assessed, as in order to minimize handling of the parasites, an attempt to purify or synchronize the cultures was not done. However, these data suggest that caution is advised when assessing *var* transcript levels and the encoded PfEMP1-binding phenotypes of individual field isolates that have been cultured even for a short length of time. It is possible that adhesion-phenotype established within the first 24 h using cellulose-filtered parasites at their late stages, represent the in vivo parasites better than non-filtered parasites. However, conclusions on parasite phenotype associations with disease outcome should be made with caution and by comparing data from identical assays with multiple isolates representing each outcome investigated.

## Conclusions

Cellulose column filtration of parasitized blood seems to be an inexpensive, rapid and effective method for improving cultivation of *P. falciparum* field isolates. However, caution is advised when assessing phenotype and genotype of cultured parasites assumed to represent the in vivo infection.

## Additional files

**Additional file 1.** Patient characteristics from the children’s ward at Korogwe District Hospital.

**Additional file 2.** Parasitaemia of ring and late stage infected erythrocytes.

**Additional file 3.** Multiplication rate.

**Additional file 4.** Transcript levels of var subtype.

## Abbreviations

PfEMP1: *Plasmodium falciparum* erythrocyte membrane protein 1
*Pfmsp2*: *Plasmodium falciparum* merozoite surface protein 2
CIDR: cysteine-rich interdomain region
EPCR: endothelial protein C receptor
RDT: rapid diagnostic test
CPDA: citrate phosphate dextrose adenine
PBMC: peripheral blood mononuclear cell
PBS: phosphate buffered saline
IE: infected erythrocyte
