# Supplementary material for: Cellulose filtration of blood from malaria patients for improving ex vivo growth of Plasmodium falciparum parasites
Source: Malar J. 2017 Feb 10;16:69. doi: 10.1186/s12936-017-1714-2 (PMC5301330; doi:10.1186/s12936-017-1714-2)
Supplement: Supplementary file 1 — Additional file 1. Patient characteristics from the children’s ward at Korogwe District Hospital. [file 12936_2017_1714_MOESM1_ESM.pdf]

Additional file 1. Patient characteristics from the children's ward at Korogwe District Hospital.

ID	Hb (g/dL)	RD	Blantyre coma score	Parasitaemia%
4636	3.1	No	5	61.15
4638	8	No	5	44.45
4647	6.1	No	5	4.30
4648	4.8	No	5	3.10
4649	5.3	No	5	0.28
4651	11.7	No	5	2.20
4656	9.3	No	5	0.09
4658	8.3	No	5	34.55
4659	8.8	No	5	4.10
4660	10.7	No	5	6.20
4661	5.5	No	5	0.02
4662	9.9	No	5	3.70
4663	9.4	No	5	15.70

RD: Respiratory distress
