# Supplementary material for: Cellulose filtration of blood from malaria patients for improving ex vivo growth of Plasmodium falciparum parasites
Source: Malar J. 2017 Feb 10;16:69. doi: 10.1186/s12936-017-1714-2 (PMC5301330; doi:10.1186/s12936-017-1714-2)
Supplement: Supplementary file 2 — Additional file 2. Parasitaemia of ring and late stage infected erythrocytes. [file 12936_2017_1714_MOESM2_ESM.pdf]

Additional file 2. Parasitaemia of ring and late stage infected erythrocytes in %.

Days post admissior		0		1		2		3		4		5	
**ID**	**Cellulose**	**LS**	**R**	**LS**	**R**	**LS**	**R**	**LS**	**R**	**LS**	**R**	**LS**	**R**
4636	No cellulose	0	0.3	0.1	0.25	0.22	0.41	0.42	1.2	1.17	2.4	Died	
	Cellulose	0	0.24	0.21	0.06	0.1	1.04	1.1	0.45	0.44	5.3	Died	
4638	No cellulose	0	0.2	0.06	0.19	0.1	0.21	0.2	0.4	0.42	1.2	Died	
	Cellulose	0	0.27	0.25	0.1	0.09	1.23	1.26	0.44	0.43	6.5	Died	
4658	No cellulose	0	0.18	0.04	0.15	0.06	0.2	0.14	0.25	0.23	0.64	0.71	1.2
	Cellulose	0	0.31	0.3	0.05	0.03	1.43	1.51	0.14	0.15	6.98	7.41	0.72
4659	No cellulose	0	0.22	0.05	0.21	0.18	0.23	0.25	0.76	0.91	1.31	1.26	4.36
	Cellulose	0	0.19	0.183	0.01	0.01	0.91	0.92	0.04	0.04	4.55	4.61	0.2
4660	No cellulose	0	0.3	0	0.27	0.25	0.1	0.2	1.13	1.2	0.93	Died	
	Cellulose	0	0.26	0.14	0.11	0.12	0.68	0.71	0.63	0.6	3.33	Died	
4662	No cellulose	0	0.17	0	0.16	0.11	0.04	0.05	0.53	0.52	0.26	Died	
	Cellulose	0	0.24	0.22	0.1	0.12	1.2	1.14	0.61	0.6	5.62	5.6	2.44
4663	No cellulose	0	0.16	0.14	0.03	0.02	0.64	0.63	0.12	0.11	3.09	3.11	0.47
	Cellulose	0	0.28	0.25	0.11	0.13	1.23	1.25	0.64	0.66	6.2	6.25	3.4

Parasites were cultured until five days after blood sampling or the appearance of dead parasites, where the latter was defined as “Died” and the parasitaemia was not determined.
