# Supplementary material for: Cellulose filtration of blood from malaria patients for improving ex vivo growth of Plasmodium falciparum parasites
Source: Malar J. 2017 Feb 10;16:69. doi: 10.1186/s12936-017-1714-2 (PMC5301330; doi:10.1186/s12936-017-1714-2)

Additional file 3. Multiplication rate**,** the number of ring stages observed compared to the number of late stages observed the previous day, either cellulose-filtered (+) or non-filtered parasites (-) from day 1 to day 5. The lower whisker indicates the lowest data point above Q1 minus 1.5 times IQR, the upper whisker indicates the highest datapoint below 1.5 IQR plus Q3. Values below Q1 minus 1.5 IQR and above Q3 plus 1.5 IQR are indicated as points.

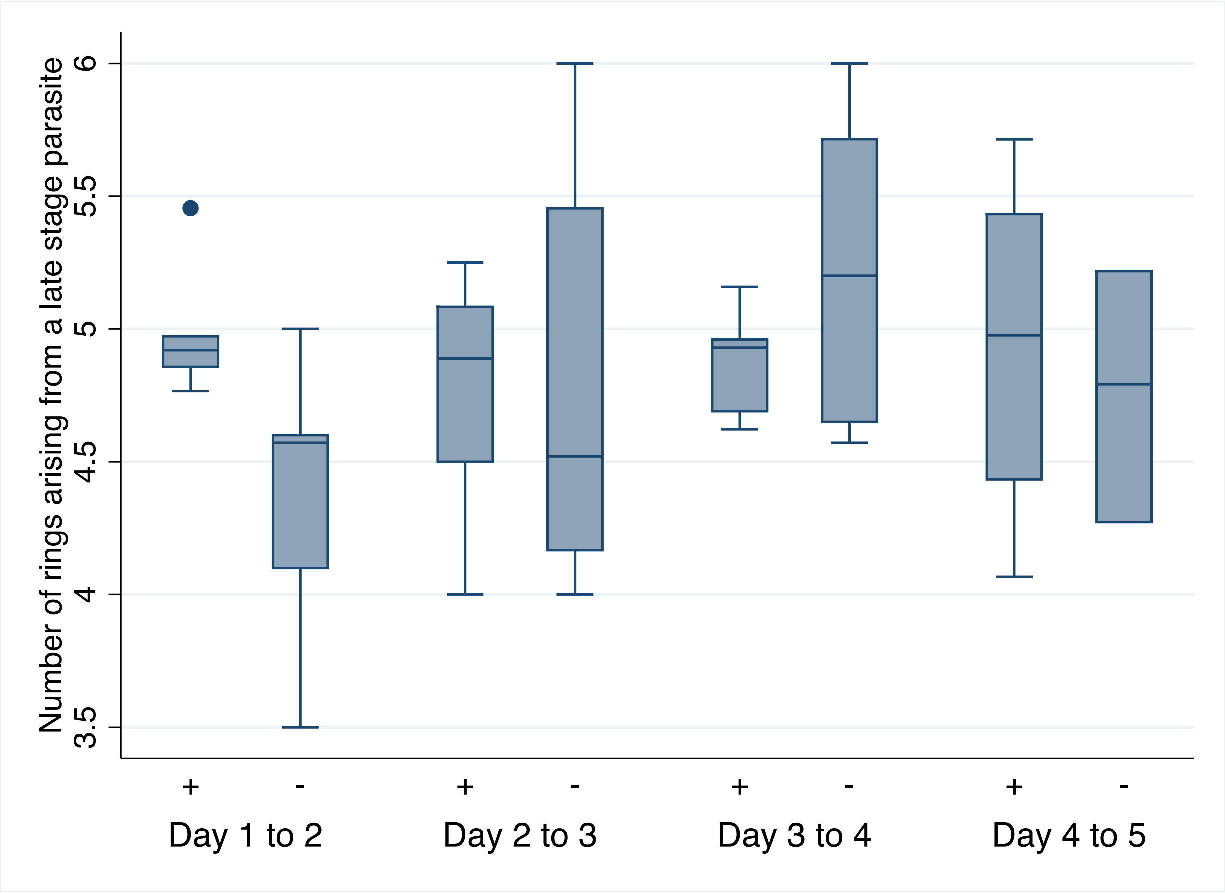

Supplement: Supplementary file 3 — Additional file 3. Multiplication rate. [file 12936_2017_1714_MOESM3_ESM.pdf]
