# Supplementary material for: Cellulose filtration of blood from malaria patients for improving ex vivo growth of Plasmodium falciparum parasites
Source: Malar J. 2017 Feb 10;16:69. doi: 10.1186/s12936-017-1714-2 (PMC5301330; doi:10.1186/s12936-017-1714-2)
Supplement: Supplementary file 4 — Additional file 4. Transcript levels of var subtype. [file 12936_2017_1714_MOESM4_ESM.pdf]

Additional file 4. Transcript levels [median Tu level (25 and 75% IQR)] of *var* subtype in *P. falciparum* parasites at Admission, after four days in culture of the cellulose-filtered and non-filtered parasites, respectively. CIDRa1.all is a summated transcript level for all CIDRα1-domain primers as described in [14]. Summated transcript level of the CIDRa1.8a and b and CIDRa1.5a and b primers were used, respectively.

Primer	Admission	Day 4 cellulose	Day 4 no cellulose
**CIDRa1.all**	**23.3**	**24.5**	**15.7**
P25	15.5	13.4	3.9
P75	56.4	99.2	50.6
**CIDRa1.1**	**1**	**3.7**	**1**
P25	1	1	1
P75	15.3	76.2	14.6
**CIDRa1.8**	**7.9**	**4.1**	**2.5**
P25	1	1	1
P75	41.3	10.7	8.2
**CIDRa1.4**	**5.2**	**4.2**	**3.1**
P25	1	1.5	1
P75	11.5	17.8	7.2
**CIDRa1.5**	**1**	**1.5**	**1.4**
P25	1	1	1
P75	2.3	4.5	3.4
**CIDRa1.6**	**1.1**	**2.3**	**1**
P25	1	1	1
P75	41.3	4.5	8.2
**CIDRa1.7**	**1.4**	**2.9**	**1.5**
P25	1	1.6	1
P75	5.1	5.3	3.2
**CIDRa3.1/2**	**1**	**1**	**1**
P25	1	1	1
P75	1.9	1	1
**CIDRd**	**1**	**1.9**	**1**
P25	1	1	1
P75	5.4	4.3	1
**var2csa**	**1.4**	**6.6**	**1.7**
P25	1	1.3	1
P75	5.3	16.6	4.1
**var3**	**3.3**	**1**	**1**
P25	1	1	1
P75	11.4	3	2.8
